# Adaptation of the online moral disengagement scale to the Turkish population and its association with empathic tendency and cyberbullying

**DOI:** 10.1186/s40359-025-02992-7

**Published:** 2025-07-11

**Authors:** Fuad Bakioğlu, Bahtiyar Eraslan Çapan, Sami Kırteke, Amir H Pakpour

**Affiliations:** 1 Faculty of Education, Department of Guidance and Psychological Counseling, Karamanoğlu Mehmetbey University, Karaman, Turkey; 2 Faculty of Education, Department of Guidance and Psychological Counseling, Anadolu University, Eskişehir, Turkey; 3 Ministry of National Education, Konya, Turkey; 4 Department of Nursing, School of Health and Welfare, Jönköping University, Gjuterigatan 5, 553 18, Jönköping, Sweden

**Keywords:** Moral disengagement, Cyberbullying, Empathic tendency, Online, Adolescent

## Abstract

**Background:**

Traditional moral disengagement is observed in daily life. However, with the increase in time spent in virtual environments, the need to also investigate online moral disengagement (OMD) is evident. In this study, we aimed to adapt the OMD Scale (OMDS) to the Turkish population and examine the relationship among OMD, empathic tendency, and cyberbullying.

**Methods:**

A total of 694 volunteering Turkish adolescents, 404 (58.2%) females and 290 (41.8%) males and with a mean age of 15.19 years (range: 14–17 years; standard deviation = 1.09), were included. Data were collected using the OMDS, Cyberbullying Scale, and Adolescent KA-Sİ Empathic Tendency Scale. The study was conducted in several stages: confirmatory factor analysis (CFA), item factor loading, item-total correlation, concurrent analysis, mediating analysis, and reliability analysis.

**Results:**

The CFA confirmed the validity of the eight-item structure OMDS. The findings also revealed a significant relationship between OMD, empathic tendency, and cyberbullying. Precisely, OMD mediated the relationship between empathic tendency and cyberbullying.

**Conclusion:**

The reliability values of the OMDS were good. Likewise, the Turkish version of the scale was also valid and reliable. OMD mediated the relationship between empathic tendency and cyberbullying.

## Introduction

In the contemporary era, technological advancements have facilitated the proliferation of online communication. Digital environments can be accessed through a range of mobile devices, including smartphones, computers, and tablets. According to the 2025 Global Overview Report, “5.56 billion individuals worldwide are connected to the internet,” and there are “77.3 million internet users in Turkey” alone [1]. Simultaneously, this rapid increase in the number of internet users every year has also led to an alarming level of cyberbullying behavior [2–4].

Cyberbullying is defined as an aggressive, deliberate, or malicious behavior of a group or individual, using virtual forms of communication (such as texts, emails, and social networking sites), repeatedly against victims who cannot defend themselves [5]. Communication using technological tools and cyberbullying behaviors are increasing, especially among adolescents. Two studies found that 23% [6] and 48% [7] of adolescents are victims of cyberbullying. Two other studies found that 21% and 79.3% of the participants were cyberbullies [8] and 34% were victims [9]. Furthermore, research has shown that cyberbullying negatively affects the mental health of its victims and is associated with depression, anxiety, stress, emotional problems, low self-esteem, and suicidal thoughts [2]. Considering the serious effects of cyberbullying on adolescents, more comprehensive studies on the causes of such behavior, how it can be prevented, and how it can be stopped are needed [10, 11].

In recent years, moral values have been identified as the main predictor of cyberbullying behaviors [12]. While moral values can help adolescents perform healthy behaviors and avoid misbehaviors [13], moral dissolution can lead to the distortion of moral standards according to self-interest and pave the way for negative behaviors [14]. Moral dissolution is defined as individuals’ cognitive tendencies to override their internal moral standards, causing them to behave unethically [15]. With the shift of communication to online environments through technological developments, discussions on the concepts of cyberbullying and online moral dissolution are becoming more common, especially among adolescents.

Online moral disengagement (OMD) occurs in online environments [16]. Individuals engaged in moral disengagement may exhibit behaviors such as posting harmfully intended messages, deliberately creating gossip, and posting embarrassing photographs in online environments [17]. Studies have shown that there is a significant positive relationship between moral disengagement and cyberbullying [12, 14, 15, 18]. Furthermore, there are several studies investigating the relationship between OMD and cyberbullying [19, 20].

One of the personality traits examined concerning cyberbullying is empathy. Empathy comprises two structures: empathic tendency and empathic skill. It is defined as the process of viewing events from the perspective of another individual, placing oneself in that individual’s shoes, accurately comprehending and experiencing that individual’s emotions and thoughts, and conveying that situation to the individual [21, 22]. Simply put, empathy is the capacity to comprehend the emotions and experiences of others. While it fosters positive social interactions, it can also impede bullying behaviors [23–26]. Emotional and cognitive empathy serve as protective factors against cyberbullying [27]. While it is asserted that a high empathic tendency reduces aggression because it allows individuals to predict the possible harm that their aggressive behavior may cause [28], low empathy is believed to be a risk factor for traditional bullying and cyberbullying [25].

### Present study

In recent years, the prevalence of cyberbullying has increased alongside the use of technological tools for communication among young people [29, 30]. According to a study, the manifestation of aggressive behavior is influenced by the interaction between individual characteristics and environmental factors [31]. The present study aimed to elucidate the factors that contribute to cyberbullying behaviors, one of the leading contributors of mental health problems, and to investigate the effects of protective and predisposing variables. Moral disengagement was considered a predisposing variable to cyberbullying, while empathic tendency was considered a protective factor. Cognitive distortions such as moral disengagement provide reasons why cyberbullying behavior is seen as normal [32–35], whereas emotional variables such as empathic tendency provide an alternative explanation for cyberbullying by being sensitive to the emotions of others. The latter has been demonstrated to have a reducing effect on bullying behavior [24, 26].

The online environment affects cyberbullying. The anonymity of the online environment allows bullying behavior to be carried out by hiding one’s identity and without the necessity of coming face-to-face with the victim [36]. The lack of direct communication with the victim, distance, and lack of visibility contribute to the use of moral disengagement strategies and the inability to be instantly informed of the emotional reactions of the victim. Emphasizing this point, research demonstrated that a lack of awareness of victims’ social-emotional cues reduces empathic tendencies and paves the way for cyberbullying [6, 37].

Studies investigating the relationship between cyberbullying and moral disengagement generally use the traditional moral disengagement scale [16], and those investigating traditional bullying and cyberbullying implement different moral disengagement strategies. To prevent cyberbullying, which is rapidly increasing in the age of technological communication, a scale is needed to measure OMD strategies. An examination of the scales related to moral disengagement in Turkey revealed that there are measurement tools for measuring traditional moral disengagement. For instance, the Turkish version of the MDS [38] aims to determine the traditional moral disengagement levels in adults, and the Collective Moral Disengagement Scale [39] aims to determine the collective moral disengagement levels in adolescents. However, no scale has been developed for determining moral disengagement behavior in online environments.

The objective of this study was to adapt the OMD Scale (OMDS) to the Turkish population and to examine the mediating role of OMD in the relationship between empathic tendency and cyberbullying. The research also aims to investigate the effect of moral disengagement and empathy in explaining cyberbullying in Turkey and to contribute to improving students’ empathic skills and preventing moral disengagement and cyberbullying.

## Methods

### Participants, procedure, and ethics

The study included 694 volunteering Turkish adolescents, comprising 58.2% (*n* = 404) females and 41.8% (*n* = 290) males, with a mean age of 15.19 years (range 14–17 years; standard deviation = 1.09). The participants were distributed across different grade levels: 37.2% in the 9th grade, 21.0% in the 10th grade, 27.2% in the 11th grade, and 14.6% in the 12th grade. To preserve the participants’ identity, the data was anonymized. In addition, the survey was designed to consider participant anonymity and confidentiality. The inclusion criteria were as follows: adolescents, voluntary participation, and secondary school students. The participants completed the questionnaire in approximately 20–25 min. The present study was conducted in accordance with the Declaration of Helsinki and was approved by the Ethics Committee of Anadolu University (number: 696630).

### Translation process

The Turkish adaptation of the OMDS followed a standard procedure [40]. First, the OMDS was translated from English to Turkish by three bilingual native Turkish translators. Second, three psychology experts consolidated the three Turkish translations into a single form. Finally, the scale was translated back from Turkish to English by two psychology experts. The final Turkish version of the scale was evaluated by two psychology experts who compared the original and translated forms.

### Measures

#### OMDS

The OMDS [20] includes eight unidimensional items (e.g., assuming that the identity of a classmate/friend online is just a game among friends) and a 5-point Likert scale (where 1 = strongly disagree and 5 = strongly agree). A confirmatory factor analysis (CFA) of the scale showed that all fit indices were good (χ^2^ = 378.526, df = 184, χ^2^/df = 2.057, *p* =.000; root mean square error of approximation [RMSEA] = 0.035, weighted root mean square residual = 0.970, comparative fit index [CFI] = 0.950) [20].

### Cyberbullying scale (CBS)

The CBS [41] has 45 unidimensional items (e.g., have you ever been assaulted to tape the assault and hang it on the internet? ) and a 4-point Likert scale (where 0 = never and 3 = always). Cronbach’s α (α = 0.82) and test-retest reliability coefficient (0.74) of the scale were found to be good. Similarly, Cronbach’s α (α =. 90) and test-retest reliability coefficient (0.90) of the Turkish version [42] of the scale were also found to be good. Overall, Cronbach’s α (0.89) in the present study was good.

### Adolescent KA-Sİ emphatic tendency scale (ETS)

The ETS [43] is a two-dimensional 13-item scale (e.g., emotional empathy: I get upset when I see someone ostracized by their friends; cognitive empathy: when something bothers my friends, even if they do not say it, I can understand their behavior) and includes a 4-point Likert scale (where 1 = not suitable for me at all and 4 = totally suitable for me). Cronbach’s α (α = 0.82) and test-retest reliability coefficient (0.74) of the scale were found to be good. Similarly, Cronbach’s α (emotional empathy α = 0.82; cognitive empathy α = 0.82) and test-retest reliability coefficient (emotional empathy = 0.73, cognitive empathy = 0.69) of the Turkish version of the scale were also found to be good. Overall, Cronbach’s α (emotional empathy α = 0.85; cognitive empathy α =. 82; total α = 0.84) in this study was good.

### Data analysis

In the present study, the OMDS was adapted to the Turkish population in several stages. First, a CFA using diagonally weighted least squares (DWLS) was performed to determine the validity of the Turkish version of the scale. The DWLS estimator was used in CFA due to the ordinal structure of the data. When evaluating the CFA results, we checked whether the fit indices were above the acceptance value (e.g., CFI, goodness-of-fit index [GFI], incremental fit index [IFI], normed fit index [NFI], and Tucker–Lewis index [TLI] ≥ 0.90; RMSEA and standardized root-mean-square residual [SRMR] ≤ 0.08) [44, 45]. Item factor loads and item-total correlation values were also examined. Second, a reliability analysis of the scale was performed. Cronbach’s α, McDonald’s ω, Guttmann’s λ6, and test-retest coefficients were calculated for reliability. Third, Pearson correlation values were examined for concurrent analysis. Fourth, the mediating role of OMD in the relationship between empathic tendency and cyberbullying was examined. Bootstrapping analysis (10,000 resampling and 95% confidence intervals [CIs]) was performed. The study data were analyzed using SPSS Statistics 28.0, JASP 16.0, and Amos Graphics 23.

## Results

First, CFA was performed to determine the validity of the Turkish version of the OMDS. All GFIs of the scale were found to be good (χ^2^ = 72.929, df = 20, χ^2^/df = 3.65, *p* =.00; CFI = 0.974, IFI = 0.974, non-NFI [NNFI] = 0.963, TLI = 0.963, RMSEA = 0.062, SRMR = 0.072). The item factor load values ranged from 0.426 to 0.930, and the item-total correlations ranged from 0.368 to 0.852 (Table 1).

**Table 1:** Statistics on the items of the OMD scale

Item	Factor Loading	Item-total correlation	M (SD)	Skewness	Kurtosis
Item 1	0.930	0.852	1.61 (0.71)	0.88	0.04
Item 2	0.457	0.373	1.80 (0.80)	0.71	− 0.17
Item 3	0.650	0.555	1.57 (0.95)	1.76	1.40
Item 4	0.874	0.732	1.43 (0.76)	1.51	0.87
Item 5	0.897	0.758	1.37 (0.72)	1.74	1.78
Item 6	0.441	0.381	1.68 (0.87)	1.23	1.02
Item 7	0.426	0.368	1.65 (0.83)	1.25	1.27
Item 8	0.870	0.712	1.32 (0.69)	1.92	1.87

* All factor loadings were significant at 0.001. *M* = Mean; *SD* = Standard deviation

Second, the reliability of the scale was calculated. Results of the reliability analysis showed that Cronbach’s α (α = 0.84, 95% CI = 0.82, 0.86), McDonald’s ω (ω = 0.84, 95% CI = 0.82, 0.86), and Guttmann’s λ6 (λ6 = 0.88, 95% CI = 0.87, 0.89) values were satisfactory. To further enhance the scale’s reliability, the same scale was applied to a group of 60 people with an interval of 3 weeks, and the test-retest coefficient was calculated. The test-retest coefficient (*r* =.84, 95% CI = 0.73, 0.91) was also found to be satisfactory.

Third, concurrent analysis was performed. Pearson correlation coefficient values were calculated between cyberbullying, OMD, and empathic tendency. The analysis revealed a significant negative correlation between empathy and moral disengagement (empathy total *r* = −.35, 95% CI = − 0.41, − 0.28, *p* <.001; emotional empathy *r* = −.31, 95% CI = − 0.38, − 0.24, *p* <.001; cognitive empathy *r* = −.29, 95% CI = − 0.36, − 0.22, *p* <.001) and cyberbullying (*r* = −.30, 95% CI = − 0.23, − 0.37, *p* <.001) and a significant positive correlation between moral disengagement and cyberbullying (*r* =.36, 95% CI = 0.30, 0.43, *p* <.001).

**Fig. 1:**
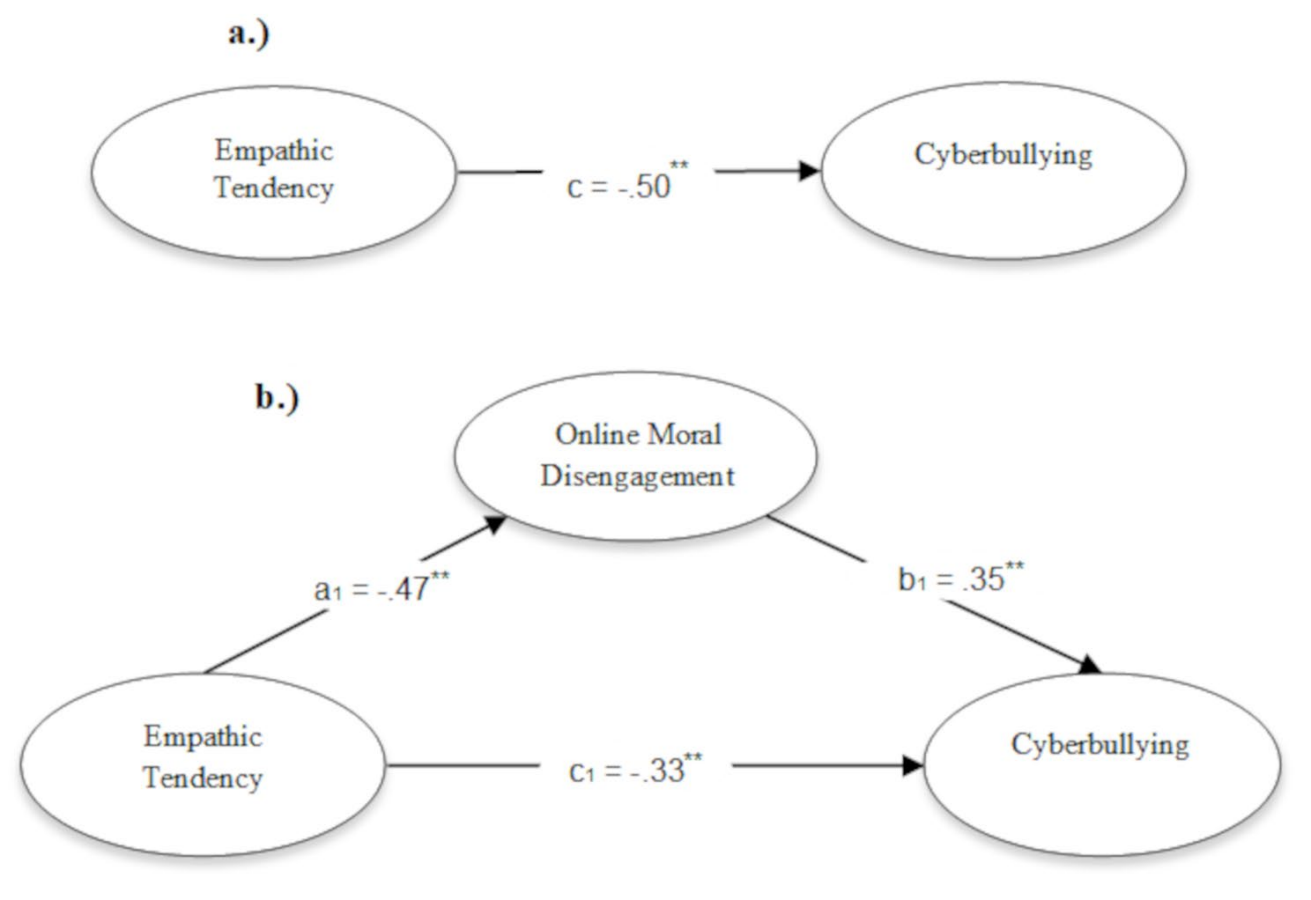
Mediated outcomes on cyber bullying showing indirect effects of empathic tendency through online moral disengagement

Finally, the mediating role of OMD in the relationship between empathic tendency and cyberbullying was examined (Fig. 1). Examination of the direct effects in the analysis showed that empathic tendency negatively predicted OMD (*B* = − 0.47, 95% CI = − 0.56, − 0.39, *p* <.001) and cyberbullying (*B* = − 0.33, 95% CI = − 0.49, − 0.20, *p* <.001), while OMD positively predicted cyberbullying (*B* = 0.35, 95% CI = 0.23, 0.53, *p* <.001). Furthermore, bootstrapping analysis was applied to determine the significance of the indirect effects; after the analysis, 10,000 resamples and 95% confidence intervals were determined. Examination of the indirect effects showed that OMD significantly mediates the relationship between empathy (value = − 0.17, 95% CI = − 0.25, − 0.11) (Table 2). All GFIs of the mediation model were found to be good (χ^2^ = 11.683, df = 6, χ^2^/df = 1.94, *p* =.00; CFI = 0.995, IFI = 0.995, NNFI = 0.989, TLI = 0.987, RMSEA = 0.037, SRMR = 0.013).

**Table 2:** Empathic tendency predicts cyberbullying through online moral disengagement

Variable	β	SE		*p*	LL (95%CI)	UL (95%CI)
Direct effect						
Empathic tendency → OMD	-0.47	0.043		0.001	− 0.558	− 0.389
OMD → Cyberbullying	0.35	0.077		0.001	0.226	0.526
Empathic tendency → Cyberbullying	-0.33	0.074		0.001	− 0.488	− 0.201
*Bootstrapping analysis for Indirect effect*						
Empathic tendency → OMD → Cyberbullying	− 0.17	0.038		0.001	− 0.253	− 0.106

Bootstrap sample size = 10,000; OMD, online moral disengagement; LL, lower limit; UP, upper limit; ^a^

## Discussion

In this study, we adapted the OMDS [20] to the Turkish population and investigated the relationship among OMD, empathic tendency, and cyberbullying. The study data were collected using the Turkish form of the scale, which was translated from English to Turkish. The research was conducted in several stages, and the findings and discussion of each stage are presented below.

First, a CFA was conducted to determine the validity of the OMDS. The analysis showed that all GFIs of the scale, which consists of 8 items and 1 dimension, were good. The fit values of the Turkish version of the scale were also similar to those of the original version [20]. The factor loadings of the Turkish version ranged from 0.426 to 0.930, and the item total correlations ranged from 0.368 to 0.852. The item factor loadings are expected to be ≥ 0.40 [46–48]. Overall, the analysis confirmed the validity of the Turkish version of the scale.

Second, Cronbach’s α (α = 0.84), McDonald’s ω (ω = 0.84), Guttmann’s λ6 (λ6 = 0.88), and test-retest coefficients (*r* =.84) were calculated to determine the reliability of the scale. A reliability coefficient of ≥ 0.70 means that the scale has high reliability [49], and in this study, Cronbach’s α, McDonald’s ω, and Guttmann’s λ6 values were > 0.70. Furthermore, the test-retest values obtained indicated that the scale produces stable measurements.

Third, a concurrent analysis was performed, which revealed a significant negative correlation between empathic tendency and OMD and cyberbullying and a significant positive correlation between OMD and cyberbullying. These findings are consistent with those of previous studies that reported a negative correlation between empathic disposition and moral disengagement [50–52]. Empathy, the cognitive and emotional understanding of another’s feelings, is negatively correlated with moral disengagement; in fact, it is distinct from moral disengagement. Precisely, while moral disengagement allows individuals to justify their aggressive behaviors from their own perspective [33, 53], empathy enables individuals to consider the situation from the other person’s point of view, take responsibility for their own actions, and act more humanely [23, 26, 34, 54]. Individuals with a high tendency toward empathy are aware of how their actions and words can impact others. High levels of empathy can reduce aggression, as individuals can anticipate the potential harm that aggressive behavior may cause to others [28].

As mentioned, the present study found a negative correlation between empathy and cyberbullying, in line with previous studies [55, 56]. Low empathy is associated with cyber aggression [33]; it prevents individuals from taking responsibility and feeling guilty for their behavior, thereby increasing cyberbullying [34]. However, some studies suggested that cyber aggression is more closely related to emotional empathy than cognitive empathy and that it tends to increase as emotional empathy decreases [14, 57].

Research has consistently shown a positive correlation between OMD and cyberbullying [50]. Several studies have identified moral disengagement as a predictor of cyberbullying [33, 57, 58]. Reportedly, cyberbullying perpetrators often view their behavior as a joke rather than something malicious [58–60]. With the ever-increasing number of internet users every day, both in the world and in Türkiye, the increase in cyberbullying cases is also more likely to occur [1].

Fourth, OMD plays a mediating role in the relationship between empathic tendency and cyberbullying, consistent with the results of a previous study [51], which revealed that moral disengagement plays a mediating role between empathy and cyberbullying. Another study also supports this finding by suggesting that aggressive behaviors are mediated by empathy and moral disengagement [61]. Research suggests that increased empathic tendency is associated with decreased moral disengagement and cyberbullying [32–35]. Both empathy and moral disengagement play important roles in aggressive behavior, with empathy serving as the foundation for moral behavior. Researchers emphasize that adolescents spend most of their time in the virtual environment, which limits their direct social interactions and reduces opportunities to develop social and cognitive skills [62]. In addition, researchers argue that due to less developed empathy and moral development, adolescents may take advantage of the online environment, leading to cyberbullying [63].

Despite its strengths, the present study has several limitations. First, the scales used in the study were based on self-reports, which can lead to response bias. Second, due to the study’s cross-sectional design, it is difficult to draw causal conclusions. It would be more appropriate to examine causality-effect relationships through experimental and longitudinal studies. Third, data for the study was collected from a limited number of Turkish adolescents; therefore, its findings may not be generalizable to a larger Turkish adolescent population.

## Conclusion

Based on the analyses conducted, it can be concluded that the Turkish version of the OMDS is both reliable and valid. Mental health professionals and researchers can use the OMDS to study OMD in Turkey. The OMDS is suitable for use in studies including the general population as well as the clinical population with cyber addiction.

## Data Availability

The data that support the findings of this study are available from the first author (FB) upon reasonable request.

## Abbreviations

CBS: Cyberbullying Scale
CFA: Confirmatory Factor Analysis
CFI: Comparative Fit Index
Cis: Confidence Intervals
DWLS: Diagonally Weighted Least Squares
ETS: Adolescent KA-Sİ Emphatic Tendency Scale
GFI: Goodness-of-Fit Index
IFI: Incremental Fit Index
NFI: Normed Fit Index
OMDS: Online Moral Disengagement Scale
RMSEA: Root Mean Square Error of Approximation
SRMR: Standardized Root Mean Square Residual
TLI: Tucker–Lewis Index
